# Fullerene Nanoarchitectonics with Shape-Shifting

**DOI:** 10.3390/ma13102280

**Published:** 2020-05-15

**Authors:** Katsuhiko Ariga, Lok Kumar Shrestha

**Affiliations:** 1 International Center for Materials Nanoarchitectonics (WPI-MANA), National Institute for Materials Science (NIMS), 1-1 Namiki, Tsukuba 305-0044, Japan; 2 Graduate School of Frontier Sciences, The University of Tokyo, 5-1-5 Kashiwanoha, Kashiwa, Chiba 277-8561, Japan

**Keywords:** fullerene, interface, nanoarchitectonics, self-assembly, shape-shift

## Abstract

This short review article introduces several examples of self-assembly-based structural formation and shape-shifting using very simple molecular units, fullerenes (C_60_, C_70_, and their derivatives), as fullerene nanoarchitectonics. Fullerene molecules are suitable units for the basic science of self-assembly because they are simple zero-dimensional objects with only a single elemental component, carbon, without any charged or interactive functional groups. In this review article, self-assembly of fullerene molecules and their shape-shifting are introduced as fullerene nanoarchitectonics. An outline and a background of fullerene nanoarchitectonics are first described, followed by various demonstrations, including fabrication of various fullerene nanostructures, such as rods on the cube, holes in the cube, interior channels in the cube, and fullerene micro-horns, and also a demonstration of a new concept, supramolecular differentiation.

## 1. Introduction

There are social demands for innovations to solve various problems in energy production and storage [1,2], environmental protection and remediation [3,4], biomedical treatments [5,6], and sophisticated device innovations [7,8]. The primary approach of science and technology to answer these demands is the development of functional materials systems. Having good materials is one of the most necessary matters for the future development of human society.

Development of good materials systems is roughly divided into two steps. One of them is the synthesis of materials. This step includes organic synthesis of various chemicals [9,10,11], synthesis of functional polymers [12,13], and preparation of inorganic nano-materials [14,15,16] with their intrinsic high functions. Further materialization of these fundamental components into function-combined and/or function-advanced hybrids and composites [17,18] is also important. Further steps for materials development are structure regulations and construction of functional materials using synthesized molecular/nano units [19,20]. Micro-fabrication and nano-fabrication have brought innovations in materials structuring in recent decades [21,22]. In addition to various lithographic techniques, many kinds of nano-technological advancements enable us to observe, evaluate, and manipulate nano-scale objects, even including atoms and molecules.

Nano-technological procedures definitely lead to certain innovations in materials fabrication, but conventional chemical processes, such as self-assembly/self-organization [23,24], also have unavoidable contributions. Unlike nano-technology techniques under selected conditions, self-assembly processes can be applied universally to a wide range of molecular/nano units. Self-assembly to create specific materials from simple molecular units is a superior way to convert molecular-structure information to material-level information [25]. For example, well-defined structural regulation of one-dimensional nano-tubes can be done through self-assembly of molecular units with well-designed interactive structures [26]. Extended supramolecular π-systems with specific color emissions are fabricated by self-assembly of borondipyrromethene (BODIPY) derivatives [27]. Because peptide molecules have structure-designable natures and high hydrogen bonding capabilities, they often become building blocks for self-assembly that are appropriate for materials fabrication in biomedical applications [28,29]. Recently, instructed assembly as self-assembly with a non-equilibrium process has been used for controls of living cell fates [30]. Although some unsolved problems remain in the basic science of self-assembly [31], self-assembling processes are widely used for the fabrication of functional materials with designed molecular units [32,33].

**Figure 1:**
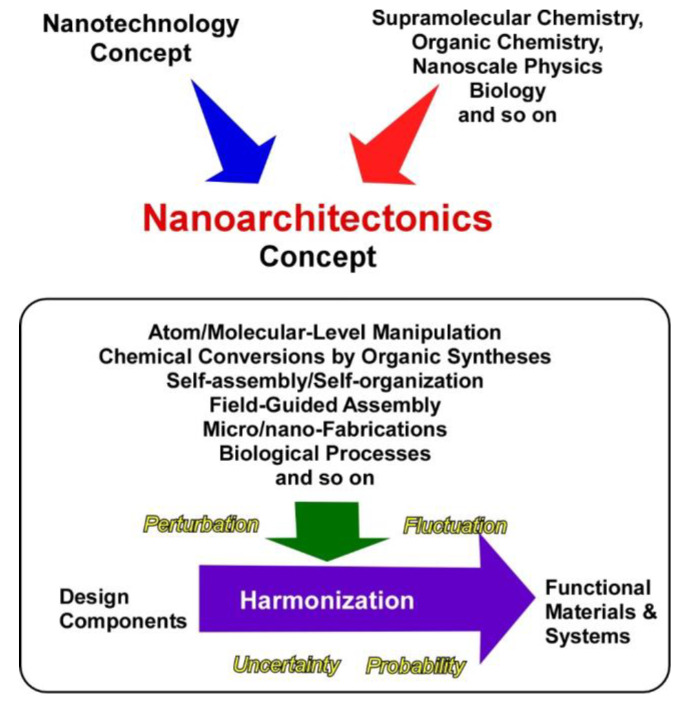
Outline of the nanoarchitectonics concept for the fabrication of functional materials and systems from molecular/nano units.

These two main research flows, developments in nano-technology and self-assembly in supramolecular chemistry, should be unified into one concept to fabricate functional materials systems from molecular/nano units with sufficient understanding of nano-level science. This task is assigned to an emerging concept, nanoarchitectonics (Figure 1) [34,35], as initially proposed by Masakazu Aono [36,37]. Nanoarchitectonics unifies nano-technology with the other scientific disciplines, such as supra-molecular chemistry, organic chemistry, nano-scale physics, and biology to architect functional materials systems from nano units. In the nanoarchitectonics process, functional materials are constructed with well-designed molecular/nano units through the combination and/or selection of various processes, including atom/molecular-level manipulation, chemical conversions by organic syntheses, self-assembly/self-organization, field-guided assemblies, micro/nano-fabrications, and biological processes [38,39]. The nanoarchitectonics concept mainly works on structural designs, but processes on functional designs are often included. Because this conceptual definition is rather ambiguous and acceptable for various materials systems, the nanoarchitectonics concept can be utilized for various research targets, such as materials synthesis [40,41], structural fabrication [42,43], catalysts [44,45], energy production and storage [46,47], environmental protection and remediation [48], sensors [49,50], sophisticated devices [51,52], biological investigations [53,54], and biomedical applications [55,56].

Unlike materials production in the macroscopic scale, materials architectonics in the nanoscopic scale cannot be simply done. In the nano-scale region, various uncertainties and complex natures, including thermal fluctuations, statistical distributions, quantum effects, and complicated mutual component interactions have unavoidable influences in materials production processes. Fabrication of functional materials is not simply explained by the summation of individual effects, and harmonization of various events is rather necessary for the synthesis of functional materials [57]. Therefore, multiple steps and non-equilibrium processes can be included in materials production. The latter features are advantageous for fabrication of hierarchic structures [58], which cannot be easily achieved by conventional self-assembling processes.

In this short review article, we pick up fullerene molecules (C_60_ and C_70_) as molecular units for the nanoarchitectonics processes to demonstrate high fabrication capabilities with structural variety from very simple units. Fullerene molecules and their derivatives are known as attractive structural units for electronic device systems [59] and solar cells [60]. In addition to their functional advantages, fullerene molecules are suitable units for the basic science of self-assembly. They are basically simple zero-dimensional objects with only a single elemental component, carbon, without any charged or interactive functional groups. In this review article, self-assembly of fullerene molecules and their shape-shifting are introduced as fullerene nanoarchitectonics. An outline and a background of fullerene nanoarchitectonics are first described, followed by various demonstrations, including fabrication of various structures, such as rods on the cube, holes in the cube, interior channels in the cube, and micro-horns, and also demonstration of a new concept, supramolecular differentiation.

## 2. Outline of Fullerene Nanoarchitectonics: Assembly and Shape-Shifts

Basic strategies for fullerene nanoarchitectonics with assembly and shape-shifts are briefly explained in this section. One of the key terms of these strategies is interface. Nanoarchitectonics processes, including self-assembly and dynamic functions at interfaces, have several specific features, such as restricted motional freedom, anisotropic structures, highly enhanced molecular interaction, and coupling of macroscopic dynamic motion and molecular functions [61,62,63]. Recent examples also show the importance of interfacial environments for various properties and functions as seen in surface domain controls through self-assembly of semi-fluorinated alkanes and related molecules [64], interface-regulated photoinduced motions of molecular arrays [65], the photocatalytic transformation of organic compounds upon surface complexation [66], heterogeneous low-temperature catalytic reactions upon surface protonics [67], and regulation of interfacial water for function design of polymeric biomaterials [68].

**Figure 2:**
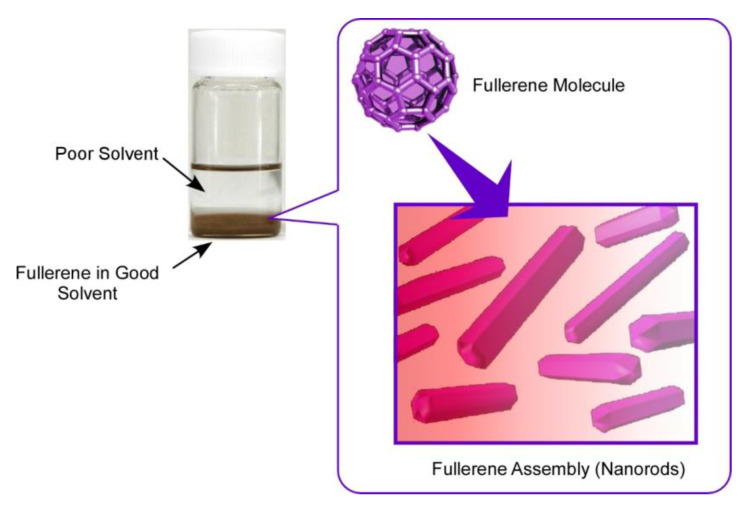
Liquid–liquid interfacial precipitation (LLIP) method for the fabrication of self-assembled structures with specific shapes.

In the case of fullerene nanoarchitectonics, the interfacial process, so-called liquid-liquid interfacial precipitation (LLIP), has been especially used for fabrication of self-assembled structures with specific shapes (Figure 2) [69]. Although several methods for the fabrication of fullerene-based assembled materials, including slow evaporation of fullerene solutions, template synthesis, and vapor depositions, have been reported, the LLIP method has a versatile nature in fabrications of dimension-controlled fullerene assemblies from nano-scale to micro-scale. As a pioneer in fullerene assembly by the LLIP method, Miyazawa and co-workers have demonstrated mainly one-dimensional whiskers, rods, and tubes in well-regulated structural dimensions [70,71]. Not limited to typical one-dimensional structures, the LLIP method can be expanded to the fabrication of two-dimensional nanosheets, three-dimensional micro-cubes, ellipsoidal structures, and their modified structures.

The LLIP method is based on the difference in solubility of fullerene molecules between contacting two solvents. For example, fullerene molecules are first dissolved in a good solvent (with higher solubility), and then a poor solvent (with lower solubility) is added quickly to make a clear interface of these two liquids. This process is usually done with static and vibration-less conditions to produce fullerene assemblies with unified shapes. Shapes of the resulting fullerene assemblies are selected by a combination of good and poor solvents. Although the above-mentioned static-type LLIP processes are done with a rather long time (up to several days), the dynamic LLIP method described below utilized short-period processes for precipitation of fullerene assemblies with applying agitation actions, such as handshaking, gentle sonication, and vortex motion. Formation of fullerene assemblies is quite quick, even occurring within a few minutes.

At interfacial regions in the LLIP processes, the poor solvent diffused into the good solvent phase, lowering the solubility of fullerene molecules at the interfacial region. This process induces fullerene clusters (nucleus) formation, and further intermixing the solvents promotes the growth of fullerene assemblies. The LLIP processes include only simple action parameters, such as solvent combination, fullerene concentration, volume ratio of good solvent and poor solvent, temperature, and so on. Tuning of these parameters creates lots of possibilities to produce fullerene assemblies with various shapes and sizes according to our design and sometimes with unexpected surprises.

As mentioned in the following sections, exposing the formed fullerene assemblies to selected solvents can induce shape modification of the assemblies through surface dissolution and re-formation of another assembling structure. In addition, selective etching of the fullerene assemblies to make holes and channels is possible using some kinds of reagents, such as amine derivatives. These processes would work for shape-shifting of the preformed fullerene assemblies.

## 3. Examples of Fullerene Nanoarchitectonics: Assembly and Shape-Shifts

### 3.1. Rods on Cube

**Figure 3:**
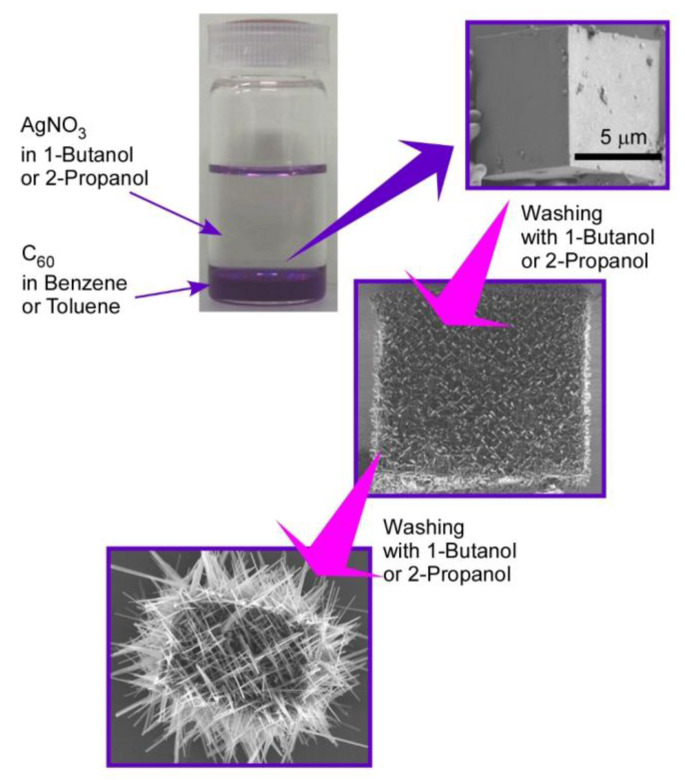
Hierarchic rods-on-cube structures of C_60_ assembly fabricated through shape-shifting from micro-cube structures prepared with C_60_ and AgNO_3_ via the LLIP method.

Hierarchic rods-on-cube structures of C_60_ assembly, where numerous rods attach to faces of the cubic structure, can be fabricated through shape-shifting from micro-cube structures prepared with C_60_ and AgNO_3_ upon the LLIP method (Figure 3) [72]. The micro-cube structures were prepared via the LLIP method using a saturated solution of C_60_ in benzene or toluene and saturated solution of AgNO_3_ in 1-butanol or 2-propanol. Micro-cubes were crystallized to sufficient size with edge lengths of 30–100 μm under static condition for 3 days to 1 week. The cubic motif was based on crystalline structures of C_60_-Ag(I) organometallic hetero-nanostructure, C_60_(AgNO_3_)_5_. A coordination network of silver(I) nitrate developed to form a zeolite-like motif and encapsulated C_60_ molecules. The fullerene–Ag(I) organometallic hetero-nanostructures were crystallized into micro-cubic structures at interfaces between benzene (or toluene) and 1-butanol (or 2-propanol).

Shape-shifting of the formed micro-cubes can be induced by exposure of the cubes with aliquots of small aliphatic alcohols, such as 1-butanol and 2-propanol. Smooth surfaces of cubes were transformed into surfaces with interpenetrated networks of rod-like crystals of pristine C_60_. Interestingly, the orientation of the rod-like structures was determined by the orientation of the different crystal planes of the original micro-cubic crystals. The first washing of the original cubes with 1-butanol kept cubic shapes where rod-like structures ran along directions parallel to the face of the micro-cubes. Growth of the needles was enhanced by further washing of the cubes upon crystallization of C_60_ through the liberation of C_60_ molecules from C_60_-Ag(I) organometallic hetero-nanostructure, C_60_(AgNO_3_)_5_. Network structural growth of rod-like structures was more extended beyond the original cube dimension after three-times washing, and further washing destroyed the cube shapes to an assembly of nano-rods.

**Figure 4:**
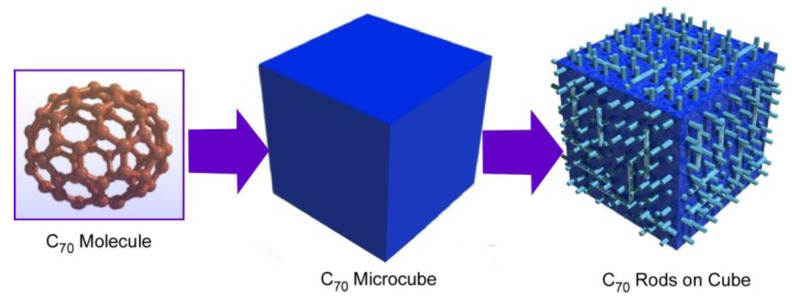
Hierarchic rods-on-cube structures of C_60_ assembly fabricated through shape-shifting from micro-cube structures prepared with C_70_ molecules.

Transformation from cubic structures to rods-on-cube architectures is possible without using additional ions, such as silver ions in C_70_ assembly (Figure 4) [73]. In the latter case, the formation of cubic structures was done with a dynamic LLIP method. The cubic assembled structures were grown at the interface between C_70_ solution in mesitylene and *tert*-butyl alcohol with the aid of ultrasound application. Washing of the resulting micro-cubes with isopropyl alcohol at room temperature induced rearrangement of assembled structures into rods-on-cube architectures where nano-rods of C_70_ grew vertically from the face.

The formed hierarchic structures, rods-on-cube structures, have indeed a high surface area because of the presence of mesoporous structures (mesopore is defined as a pore with a diameter ranging from 2 to 50 nm) on the rod moieties. This feature is advantageous as sensing membranes for external gas substances. Sensor devices using quartz crystal micro-balances (QCM) modified with the synthesized rods-on-cube C_70_ assemblies were subjected to selective detections of chemical species in gas phases. The mesoporous rods work as a sensing antenna, especially for guest molecules. In addition to the high surface area nature, easy diffusion of gas molecules within mesopores on rods resulted in a high-sensing capability. Based on strong π–π interaction with C_70_ fullerene, the prepared sensor systems exhibited selective sensitivity to aromatic guest molecules, such as benzene, pyridine, and toluene, which are considered to be experimentally toxic substances.

### 3.2. Holes in Cube

Regulated porous nano-architectures, such as mesoporous materials [74,75], metal–organic frameworks [76,77], and related nano-porous structures [78,79], have been given much attention for various applications, such as facilitating sensing, drug delivery, and energy-related applications. By contrast, materials with regulated micro-porous structures are not so common. Size-defined micro-pores are useful for trapping toxic particles, such as PM2.5 particles (PM2.5: particle matters with a diameter of 2.5 μm or less) and undesirable bio-objects, such as virus particles and bacteria. Additionally, materials with such micro-pores can find application opportunities for encapsulation, protection, transport, and release of objects in micron and submicron size, such as various biomaterials.

**Figure 5:**
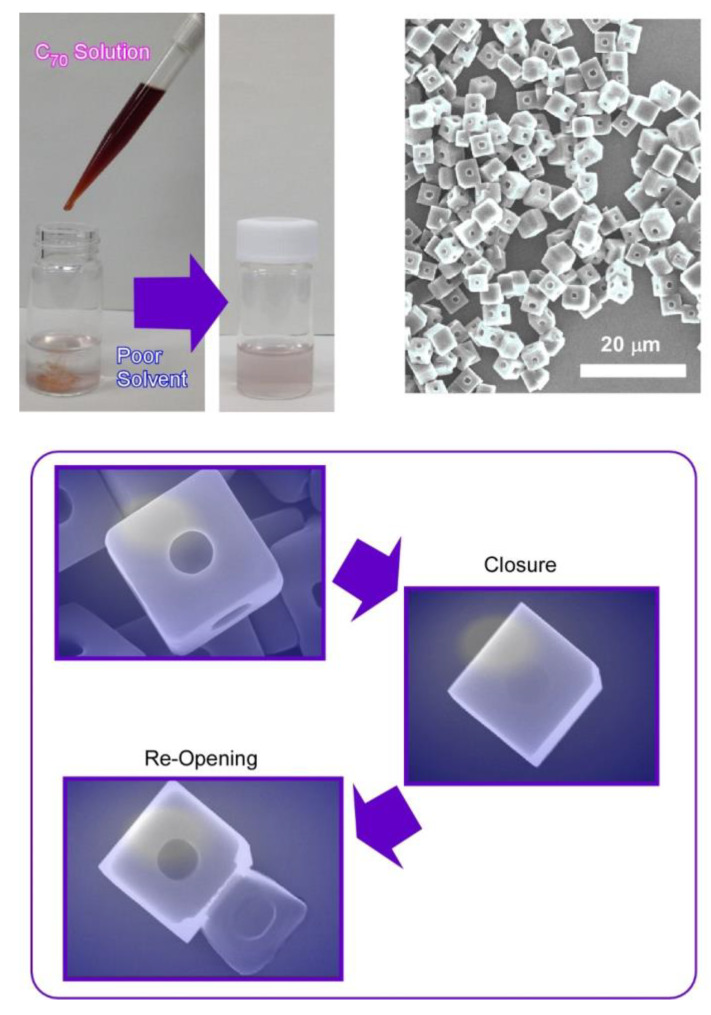
C_70_ holes-in-cube structure with one regular micro-hole at the center of every face in the cubic structures and its shape-shifts for closure and re-opening of the micro-hole.

Synthesis of micro-cubes of C_70_ molecules was performed with one regular micro-hole at the center of every face in the cubic structures (Figure 5) [80]. Shape-shifts for closure and re-opening of the micro-holes were demonstrated, together with selective trapping of micro-particles. Holes-in-cube architecture can be synthesized via the rapid LLIP method using *tert*-butyl alcohol and mesitylene as poor and good solvents, respectively. In this case, rapid addition of fullerene (C_70_) solution into the poor solvents was unusually adopted. As is the typical procedure for holes-in-cube structures, C_70_ solution in mesitylene (1 mL, 1 mg/mL) was rapidly added into poor solvent of *tert*-butyl alcohol (3 mL). The resulting mixture was then incubated for 12 h at 25 °C.

The holes formed at every face of micro-cubes can be intentionally closed though growing a thin fullerene sheet as a lid structure by adding excess C_70_ solution. Simple addition of C_70_ solution can cover almost all the open cubes. Thin films of C_70_ molecules were formed uniformly with thickness of ca. 700 nm. In addition, the resulting covered holes can be reopened by irradiation locally on the cube face using an electron beam (30 kV) for a short period (5–10 s). The thin lid layer of C_70_ was peeled off from the surface of cube faces to re-open the holes upon electron beam irradiation.

The holes-in-cube structures can discriminate carbon particles and polymer particles, although both of them have a hydrophobic nature, and their sizes are almost identical. In experiments to compare entrapment capabilities of graphitic carbon particles and resorcinol–formaldehyde polymeric resin particles, preferential capture of the former carbon particles over the latter resin particles was demonstrated based on microscopic observation by scanning electron microscopy (SEM: S-4800, Hitachi Co. Ltd. Tokyo, Japan). Most of the holes in the cubes of C_70_ molecules were occupied by the graphitic carbon particles, while entrapment of the resin particles was not so significant. The holes can preferentially entrap graphitic objects, probably due to surface π–π interaction. Although molecular-level recognition is a common subject [81,82], microscopic-level recognition for micron-sized particles has not been fully explored. The found example would open a novel category of microscopic materials recognition.

### 3.3. Inside Etching

**Figure 6:**
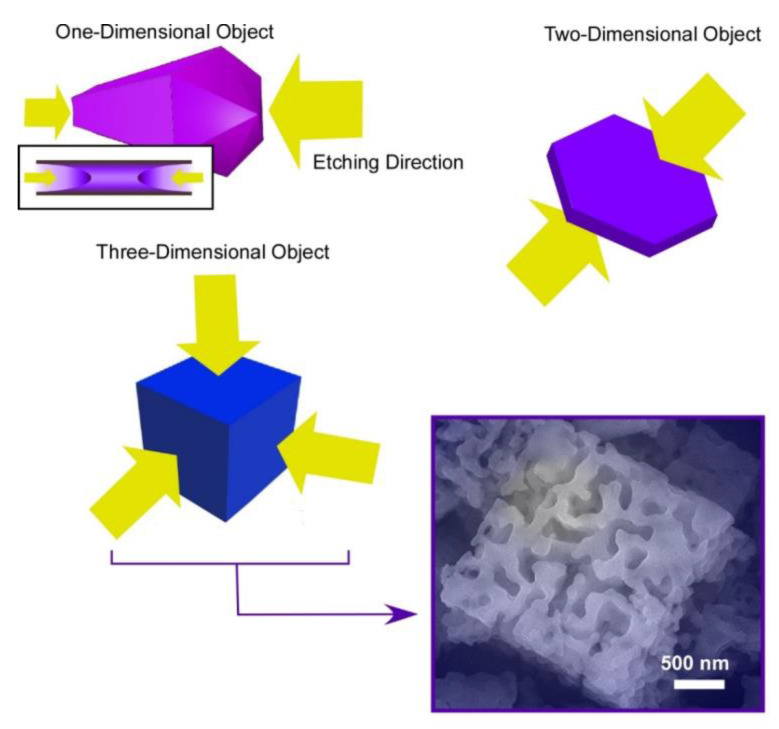
Chemical etching using ethylene diamine for preformed assembling objects in different dimensions: one-dimensional C_60_ nano-rods, two-dimensional C_60_ nano-sheets, and three-dimensional C_70_ micro-cubes.

Shape-shifting of fullerene assemblies can be chemical etching using ethylene diamine for preformed assembling objects in different dimensions: one-dimensional C_60_ nano-rods, two-dimensional C_60_ nano-sheets, and three-dimensional C_70_ micro-cubes (Figure 6) [83]. Ethylene diamine is supposed to weakly react with fullerenes to form adducts, which have increased solubility for chemical etching. The chemical etching on one-dimensional C_60_ nano-rods occurred selectively from both ends of the nano-rods. The C_60_ nano-rods were shape-shifted into their hollow-structured nano-tubes. In the case of two-dimensional C_60_ nano-sheets, the etching is proceeded selectively from upper and bottom faces of the nano-sheets. To the three-dimensional micro-cube, the etching started from six faces of the micro-cubes, inducing shape-shifting from cubes into gyroid-like objects. These etched objects bear rather hydrophilic natures with increased dispersibility to aqueous phase. In addition, the etched fullerene objects with ethylene diamine exhibited high affinity to vapors of acids, such as formic acid and acetic acid. Selective sensors for these gas objects can be prepared by immobilizing the etched fullerene objects on QCM devices.

**Figure 7:**
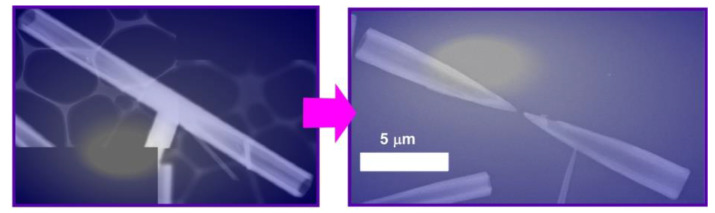
Fullerene micro-horns can be fabricated from fullerene micro-tubes of C_60_ and C_70_ mixture (these images are representative ones).

According to the post-shape-shifting procedure, fullerene micro-horns can be fabricated from fullerene micro-tubes of C_60_ and C_70_ mixture (Figure 7) [84]. In the first process, fullerene micro-tubes were fabricated via a dynamic LLIP method using C_60_/C_70_ mesitylene (good solvent) solution and *tert*-butyl alcohol (bad solvent). Shape-shifting from micro-tubes to micro-horns occurred spontaneously upon addition of mixed solvent systems of mesitylene and *tert*-butyl alcohol and slow evaporation of the solvents. The resulting micro-horns had a sharp solid tip and hollow tubular end. The length of the fullerene micro-horns (ca. 10 μm) was approximately half the length of the fullerene micro-tubes. Within the original micro-tubes, the concentration of C_70_ was decreased from the center to both ends. The addition of the mixed solvent system of good and poor solvents in a well-balanced ratio selectively etched the center of the micro-tube due to higher solubility of C_70_ over C_60_. Interestingly, the fullerene micro-horns could selectively capture hydrophilic silica particles with a couple of hundred nanometers in diameter over the other particles in a similar size, such as fullerene particles and polystyrene latex particles. Therefore, the prepared micro-horn objects would be useful for the removal and sensing of biomaterials such as virus particles.

### 3.4. Supramolecular Differentiation

As the final example, a novel concept, supramolecular differentiation, is briefly explained. Living creatures can have a complicated organization from a single cell through a biologically important process of cellular differentiation. This process supports changes of non-specialized cells to more specialized types of cells. Differentiation progresses many times during the growth of multicellular organisms in response to the transformation of organisms to complex systems of tissue and cell types. Cellular differentiation induces drastic changes to shapes of living creatures, as seen in development from eggs to tadpoles of frogs. The following example mimics such biological differentiation upon simple self-assembly of fullerene derivatives.

**Figure 8:**
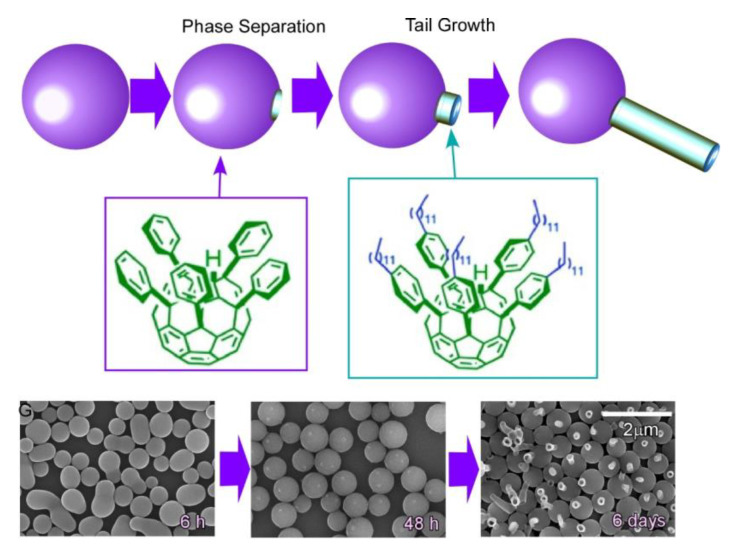
Supramolecular differentiation with the time-programmed regulation of multiple assembling processes.

Self-assembly of two kinds of C_60_ derivatives, pentakis(phenyl)fullerene and pentakis-(4-dodecylphenyl)fullerene, at liquid–liquid interfacial media (2-propanol/toluene) induces supramolecular differentiation with the time-programmed regulation of multiple assembling processes (Figure 8) [85]. In the researched system, egg-like spherical assemblies were initially resulted at the liquid–liquid interface, and mixing two phases by external stimuli, such as gentle sonication, led to the growth of tails from the original eggs to form tadpole-like structures. The processes depend on the formation of phase-separated domains of pentakis-(4-dodecylphenyl)fullerene on the surface of the sphere made of pentakis(phenyl)fullerene. Differentiation to supramolecular tadpoles upon growth of tales made of pentakis-(4-dodecylphenyl)fullerene are only possible by mixing after the formation of phase-separated domains. The observed supramolecular differentiation could be regarded as an analogue of embryonic development in the field of material science.

## 4. Conclusions

This short review article introduces several examples of self-assembly-based structural formation and shape-shifting with using very simple molecular units, fullerenes (C_60_, C_70_, and their derivatives), as fullerene nanoarchitectonics. Even though using these unit components is so simple (basically single atom composition and zero-dimension), huge varieties of assembling objects can be obtained at liquid–liquid interfaces upon changing simple conditions, such as solvent compositions, concentrations, temperatures, and additional processes such as sonication. In addition, post-treatments, such as solvent washing and chemical etching, are useful for the production of hierarchic structures and integrated morphologies. It should be noted that all the processes can be done on a lab bench. Any expensive facilities such as lithography apparatuses and specific conditions such as high vacuum, ultra-low temperatures, and extremely clean conditions are not necessary. Molecular abilities for self-assembly and self-organization are capable of forming various architectures, even with high hierarchies. More advanced structure organization upon molecular-assembling capabilities can be commonly seen in biological systems where huge kinds of molecules are spontaneously organized into sophisticated structures under ambient conditions. The latter biological systems can be regarded as an ideal model for materials science and nanoarchitectonics. From this viewpoint, novel challenges like supramolecular differentiations have to be more promoted.

In this review article, we just presented nanoarchitectonics examples of mainly simple fullerene derivatives. However, many experimental and theoretical approaches revealed various possibilities in functions and dynamic properties, together with the functionalization of fullerenes and their controlled assemblies [86,87,88,89,90]. Expanding the strategies described here to these wider candidates would create huge opportunities for function developments.

Although this review article shows rather limited application examples, fullerenes and their assemblies have various functional possibilities, as seen in bio-related applications and light energy conversion [91,92,93,94]. Possibilities in the practical application of nanoarchitected fullerene materials should be extended to a wider range of research fields, including energy, the environment, devices, and biomedicals.
